# Bayesian Modeling for Nonstationary Spatial Point Process via Spatial Deformations

**DOI:** 10.3390/e26080678

**Published:** 2024-08-11

**Authors:** Dani Gamerman, Marcel de Souza Borges Quintana, Mariane Branco Alves

**Affiliations:** 1 DME-Instituto de Matemática, Universidade Federal do Rio de Janeiro, Rio de Janeiro 21941-909, RJ, Brazil; marcel@dme.ufrj.br (M.d.S.B.Q.); mariane@im.ufrj.br (M.B.A.); 2 Instituto Nacional de Infectologia Evandro Chagas-FIOCRUZ, Rio de Janeiro 21040-360, RJ, Brazil

**Keywords:** spatial deformation, point process, Cox process, Gaussian process, Bayesian inference, MCMC, HMC

## Abstract

Many techniques have been proposed to model space-varying observation processes with a nonstationary spatial covariance structure and/or anisotropy, usually on a geostatistical framework. Nevertheless, there is an increasing interest in point process applications, and methodologies that take nonstationarity into account are welcomed. In this sense, this work proposes an extension of a class of spatial Cox process using spatial deformation. The proposed method enables the deformation behavior to be data-driven, through a multivariate latent Gaussian process. Inference leads to intractable posterior distributions that are approximated via MCMC. The convergence of algorithms based on the Metropolis–Hastings steps proved to be slow, and the computational efficiency of the Bayesian updating scheme was improved by adopting Hamiltonian Monte Carlo (HMC) methods. Our proposal was also compared against an alternative anisotropic formulation. Studies based on synthetic data provided empirical evidence of the benefit brought by the adoption of nonstationarity through our anisotropic structure. A real data application was conducted on the spatial spread of the *Spodoptera frugiperda* pest in a corn-producing agricultural area in southern Brazil. Once again, the proposed method demonstrated its benefit over alternatives.

## 1. Introduction

A spatial point process is a stochastic process that governs the location of events in a geographic space (Diggle [1]). A point pattern is a collection of locations in a given area and is interpreted as the realization of a point process (Illian et al. [2]). This type of pattern can be analyzed when georeferenced data are available and the exact locations of the events of interest are known; for example, the geographic coordinates of trees infected by a particular pest or the location of occurrences of a disease. Unlike the applications in geostatistics, in which the locations are fixed and the interest lies on the measurement of some random response, the very location of the occurrences is the random object in point processes. In the early literature on spatial point processes, it was common to assume that patterns were realizations of stationary and isotropic point processes, which implies that the statistical properties of the point process are invariant under spatial translations and rotations. However, these assumptions are often unrealistic, since in many cases there may be local or directional influences in the correlation structure of the spatial random process.

Several authors have explored nonstationarity and anisotropy in point processes. Häbel et al. [3] introduced anisotropic pair-potential functions based on Ripley’s *K*-function. Gabriel [4] proposed a similar extension in a spatiotemporal context. Hahn and Vedel Jensen [5] unified nonstationary model classes under a framework of hidden second-order stationary processes. Konstantinou and Särkkä [6] extended Ripley’s *K*-function to three dimensions using a pairwise interaction Markov field model to accommodate anisotropic data. Rajala et al. [7] devised a two-stage non-parametric method for quantifying geometric anisotropy. Sormani et al. [8] formulated a comprehensive framework for addressing geometric anisotropy, including directional versions of Ripley’s *K*-function, wavelet transforms, and spectral analysis, along with a method based on projecting Fry points onto the unit sphere. Martin et al. [9] introduced a new family of geometric anisotropic random fields and derived anisotropic point processes from them. Kwon et al. [10] utilized Hawkes process models, a class of self-exciting point process capturing event-triggering effects, to analyze earthquake occurrences. Dong et al. [11] devised another non-stationary spatiotemporal self-exciting point process, incorporating a neural network-based kernel to capture spatially varying triggering effects. Biard and Saussereau [12] applied a fractional Poisson process to situations where variances increase over time according to a defined power law. Roveri et al. [13] introduced a method to analyze and synthesize point patterns with adaptive density and correlations using a locally stationary point process. Most of these methodologies have the advantage of not requiring specific model assumptions. However, they have the disadvantage of not indicating what the intensity function would be if inherent anisotropy were taken into account, and they do not allow for an evaluation of the influence of covariates.

In geostatistics, various methods address nonstationarity/anisotropy in spatial modeling, such as process convolution models (Higdon et al. [14], Risser and Calder [15]), kernel/smoothing methods (Fuentes [16]), and basis functions (Pintore and Holmes [17], Stephenson et al. [18]). Spatial deformation allows the data to control the behavior of the fitted process without specifying mechanisms. Sampson and Guttorp [19] proposed a non-parametric method for estimating spatial covariance globally using a deformation technique. It maps coordinates from the geographical *G*-space to the deformed *D*-space (where stationarity holds), locating monitoring stations with multidimensional scaling and interpolating via thin plate splines. The inclusion of a non-linear deformation in the correlation function necessarily implies the loss of stationarity of the process and allows for anisotropic formulation, encompassing a wider range of models for dependence between locations. Challenges include multiple mappings in *D*-space and non-bijective predictions. Schmidt and O’Hagan [20] introduced a bi-dimensional Gaussian process to handle uncertainty in spatial deformation estimation.

This paper aims to apply spatial deformation to spatial point processes in order to handle anisotropy and nonstationarity. Challenges arise when adapting deformation to point processes based on Cox processes (see, for example, Diggle [1]), such as the problem of dealing with infinite dimensionality and the intractability of likelihood functions for non-homogeneous Poisson processes. Discretizing the *G*-space to address this issue is a common approach, involving forming partitions with constant intensities and using the centroids as representative points of each partition fraction (Moller et al. [21]; Brix and Diggle [22]; Paez and Diggle [23]). The discretization procedure also impacts the use of the deformation process (DP), which inherently operates in continuous space but is well-defined in finite dimensions in discretized space.

The Log–Gaussian Cox process is a standard model in point process analysis where the logarithmic function links an intensity function to a Gaussian process (GP). Adams et al. [24] used a logistic link for isotropic scenarios, and Gonçalves and Gamerman [25] applied a probit link for efficient sampling. Both bounded link functions require an extra parameter to account for the maximal intensity. An evaluation of the effect of different link functions on the intensity function estimation in anisotropic/nonstationary situations is also carried out in this paper.

This study presents an extended spatial Cox process for point process analysis for estimating intensity function with consideration for anisotropy and nonstationarity through spatial deformation. The novelty lies in using deformations to handle anisotropy in spatial point processes. The research employs the geometry-aware Hamiltonian Monte Carlo (HMC) method for sampling and evaluates different link functions across various scenarios. The methodology allows for data-driven guidance for estimating spatial anisotropy/nonstationarity behavior. It incorporates a multi-dimensional Gaussian process as a prior specification for the DP, adopting a fully Bayesian approach that assigns a probability distribution to spatial deformation as a latent process and quantifying estimation uncertainty. The method resolves the non-identifiability of the deformation, and notably, the proposed model can also accommodate intensity regressors.

The presentation of the paper is organized as follows: Section 2 describes the proposed Cox process, considering the spatial deformation through the multi-dimensional Gaussian process. Section 3 presents an analysis of the performance of the proposed model and estimation method, considering both synthetic and real data. Section 3.1 presents the results of simulation exercises for the comparison of the proposed anisotropic formulation to an isotropic one. Section 3.2 shows the estimation through the proposed anisotropic model compared to an isotropic formulation for the *Spodoptera fruguperda* pest data in an agricultural area of corn production in the city of Cascavel, Brazil. Section 3.3 provides favourable comparative results of our formulation against an alternative anisotropic specification. Section 4 discusses the results and indicates current and future extensions of this work.

## 2. Methodology

As previously mentioned, the well-established deformation technique involves mapping geographic coordinates from the original space (*G*-space) to a new latent space (*D*-space), ensuring stationarity and isotropy. This work employs an approach similar to previous studies, such as Schmidt and O’Hagan [20] and Morales et al. [26], where the random function d:G→D transforms original coordinates from *G*-space to the new *D*-space, following a prior *l*-variate Gaussian process

(1) $$\begin{array}{c} d|\sigma_{d}^{2},\phi ∼GP\left(h,\sigma_{d}^{2},\rho_{\phi }\right), \end{array}$$

where *h* is a prior mean function, $\sigma_{d}^{2}$ is a l×l covariance matrix and ϕ is the vector of parameters for any valid correlation function

(2) $$\begin{array}{c} Corr\left(d\left(s_{i}\right),d\left(s_{j}\right)\right)=\rho_{\phi }(∥s_{i}-s_{j}∥),\;\forall s_{i}\;\mathrm{and}\;s_{j}\in G. \end{array}$$

The covariance structure of the GP is essential for representing the behavior of the function *d*. It controls the prior warping degree of the mapping. Details on the specification of GP hyperparameters will be discussed later in the text.

The hierarchical structure for the proposed extension for the Cox process is given by

(3) $$\begin{array}{cc} Y|\lambda & ∼\mathrm{PP}(\lambda ) \end{array}$$

(4) $$\begin{array}{cc} \lambda (s) & =\lambda^{*}g\left(\mu +\beta \left(s\right)\right),\;\forall s\in G \end{array}$$

(5) $$\begin{array}{cc} \beta |\delta ,\theta ,d & ∼\mathrm{GP}(0,1/\delta ,\rho_{\theta }) \end{array}$$

(6) $$\begin{array}{cc} d|\sigma_{d}^{2},\phi & ∼\mathrm{GP}(h,\sigma_{d}^{2},\rho_{\phi }), \end{array}$$

where PP indicates a Poisson process with intensity function λ, $g^{-1}\left(\cdot \right)$ is an appropriate link function, $\lambda^{*}=\underset{s\in G}{\sup}\;\lambda \left(s\right)$ (if the supremum exists (for g(·) functions bounded to [0,1], for example *probit* or *logit* link functions, it is necessary to estimate $\lambda^{*}$ because the intensity function can take values greater than 1. For upper unbounded g(·) functions such as the exponential, $\underset{s\in G}{\sup}\;\lambda \left(s\right)=\infty$ and we can fix $\lambda^{*}=1$.)); μ is an intercept, β is a GP with mean 0, the precision is δ and the vector of parameters is θ for any valid correlation function

(7) $$\begin{array}{c} Corr\left(\beta \left(s_{i}\right),\beta \left(s_{j}\right)\right)=\rho_{\theta }(∥d\left(s_{i}\right)-d\left(s_{j}\right)∥), \end{array}$$

where $s_{i}$ and $s_{j}$ are any points, again, in *G*.

In this study, we will analyze the *probit* ($\Phi^{-1}\left(\cdot \right)$) and logarithmic link functions. The bounded range of the cumulative standard normal function Φ(·) to [0,1] requires estimating $\lambda^{*}$, whereas for the unbounded exponential function (logarithmic link), $\lambda^{*}=1$ and its estimation is not required.

The prior mean for *d* is assumed as the identity function (h(s)=s) to express prior indifference on how *D* differs from *G*. The diagonal matrix $\sigma_{d}^{2}=\mathrm{diag}\left(\sigma_{d_{11}}^{2},\;\ldots ,\;\sigma_{d_{ll}}^{2}\right)$ indicates deformation variability across dimensions. For example, when *G* denotes geographical coordinates (k=2) and *D* has the same dimension (l=2), higher/lower $\sigma_{d_{ii}}^{2}$ values allow more/less deformation along the horizontal axis (i=1) or vertical axis (i=2). Additionally, increased correlation range in (2) implies smoother deformation among nearby regions. While both β and *d* are Gaussian processes, from now on we use “GP” for β and “deformation process (DP)” for *d*, in order to clarify which process we are referring to.

The likelihood function of the PP in (3) is given by the Janossy density (see, for example, D. J. Daley [27], p. 122) and is expressed as

(8) $$L\left(\beta ,\mu ,\lambda^{*};N=n,{\left\{s_{i}\right\}}_{i=1}^{n}\right)\propto \prod_{i=1}^{n}\lambda^{*}g\left(\mu +\beta \left(s_{i}\right)\right)\exp\left\{-\int_{G}\lambda^{*}g\left(\mu +\beta \left(s\right)\right)d\nu \left(s\right)\right\},$$

with respect to a PP dominating measure with a constant intensity function, the integral corresponds to a Lebesgue integral. As previously mentioned, the likelihood function in expression (8) depends on infinite-dimensional quantities like β and *d*, which is a problem for making inferences mainly concerning the integral on all *G*-space.

When estimating model hyperparameters, fixing some or using informative priors might be necessary due to hierarchical complexity. The phenomenon being examined requires a complex, highly-structured hierarchical model, needing meticulous attention to fully comprehend and accurately elicit all its components. For $\sigma_{d}^{2}$ and ϕ, a balanced deformation degree is sought to avoid folding or a loss of anisotropy. It is well known that Φ(s)≈0 for any s<−3 and Φ(s)≈1 for any s>−3. Therefore, a reasonable strategy when using the *probit* link function is to set μ=0 and δ=0.5 to favor parameter identification in Equation (4). A Gamma prior is assigned to $\lambda^{*}$, possibly centered on some empirically-set intensity or with a non-informative specification. Under the *log* link, vague improper uniform prior for μ and non-informative Gamma prior for δ are used.

For the GP β, for the sake of simplicity, we adopted the exponential correlation function $\rho_{\theta }(∥d\left(s_{i}\right)-d\left(s_{j}\right)∥)=\exp\left(-(∥d\left(s_{i}\right)-d\left(s_{j}\right)∥)/\theta \right)$. θ follows a Gamma($a_{\theta }$, $b_{\theta }$) prior, centered on a value that produces low correlation only for higher distances in the geographic region. Defining the density of $\left(d,\theta ,\lambda^{*},\mu ,\delta ,\beta ,N=n,{\left\{s_{i}\right\}}_{i=1}^{n}|G,\sigma_{d}^{2},\phi \right)$ as the Radon–Nikodym derivative of P with respect to a suitable dominating measure W we have

(9) $$\begin{array}{c} \begin{array}{c} \pi \left(d,\theta ,\lambda^{*},\mu ,\delta ,\beta ,N=n,{\left\{s_{i}\right\}}_{i=1}^{n}|G,\sigma_{d}^{2},\phi \right)=\pi_{GP_{2}}\left(d|\sigma_{d}^{2},\phi \right)\pi \left(\theta \right)\pi \left(\lambda^{*}\right)\times \\ \pi \left(\mu \right)\pi \left(\delta \right)\pi_{GP}\left(\beta |\delta ,\theta ,d\right)\times L\left(\beta ,\lambda^{*},\mu ;N=n,{\left\{s_{i}\right\}}_{i=1}^{n}\right) \end{array} \end{array}$$

with π(θ), $\pi (\lambda^{*})$, π(μ), π(δ) denoting the prior densities described above, $\pi_{GP}\left(\beta |\ldots \right)$ and $\pi_{GP_{2}}\left(d|\ldots \right)$ as described in expressions (4) and (6), and $L(\beta ,\lambda^{*},\mu ;N=n,{\left\{s_{i}\right\}}_{i=1}^{n})$ as in expression (8). The joint posterior distribution is proportional to (9).

A usual approach to dealing with infinite-dimensional quantities β and *d* in this likelihood is to discretize the space of interest. By discretizing the geographical space into a partition $\cup_{z=1}^{Z}G_{z}=G$ where $G_{k}\cap G_{k^{\prime }}=\emptyset$ for every $k\neq k^{\prime }$, it is possible to define piece-wise constant intensities and assume that for each sub-region $G_{z}$ the centroid $s_{z}$ is a representative point. The greater the number of occurrences, the more refined the discretization could be in effectively disseminating information, leading to more accurate estimates to more adequately characterize the intensity function. Defining $\beta \left(s\right)\;:=\beta \left(s_{z}\right)\;\;\forall s\in G_{z},z=1,\ldots ,Z$, the integral in (8) can be rewritten as

$$\sum_{z=1}^{Z}\lambda^{*}g\left(\mu +\beta \left(s_{z}\right)\right)\nu \left(G_{z}\right)$$

where $\nu (G_{z})$ is the Lebesgue measure (for k=2, for example, it represents the area) of the *z*-th region; now it is possible to perform inferences for the discretized space.

Defining $y\;:=\left[y_{1},\;\ldots ,\;y_{Z}\right]$ for $y_{z}$ the total count of events in $G_{z}$, z=1,…,Z, the posterior density for the model with space discretization based on expression (9) is given by

(10) $$\begin{array}{c} \begin{array}{c} \pi \left(d_{Z},\theta ,\lambda^{*},\mu ,\delta ,\beta_{Z},|G,y,\sigma_{d}^{2},\phi \right)\propto \pi_{MVN}\left(d_{Z}|\sigma_{d}^{2},\phi \right)\pi \left(\theta \right)p\left(\lambda^{*}\right)\times \\ \pi \left(\mu \right)\pi \left(\delta \right)\pi_{MN}\left(\beta_{Z}|\delta ,\theta ,d_{Z}\right)\times \;L\left(\beta_{Z},\lambda^{*},\mu ;y\right) \end{array} \end{array}$$

where $\beta_{Z}\;:={\left[\beta \left(s_{1}\right),\;\ldots ,\;\beta \left(s_{Z}\right)\right]}_{Z\times 1}$, $d_{Z}\;:={\left[\begin{array}{ccc} d(s_{1}) & \ldots & d(s_{Z}) \end{array}\right]}_{l\times Z}$ (where l∈N is the dimension of *D*-space), $\pi_{MVN}\left(d_{Z}|...\right)$ is the matrix-variate normal density (see, for example, Morales et al. [26]), $\pi_{MN}\left(\beta_{Z}|\ldots \right)$ is the multivariate normal density with a zero mean vector and covariance matrix $\Sigma_{\beta }$ with elements ${\left[\Sigma_{\beta }\right]}_{ij}=\frac{1}{\delta }\rho_{\theta }(∥d\left(s_{i}\right)-d\left(s_{j}\right)∥)$ for i=1,…,Z and j=1,…,Z and $L(\beta_{Z},\lambda^{*},\mu ;y)$ is the likelihood function (8) by applying the discretization method, resulting in

(11) $$\begin{array}{c} L\left(\beta_{Z},\lambda^{*},\mu ;y\right)=\prod_{z=1}^{Z}{\left(\lambda^{*}\Phi \left(\beta \left(s_{z}\right)\right)\right)}^{y_{z}}\exp\left\{-\sum_{z=1}^{Z}\lambda^{*}g\left(\mu +\beta \left(s_{z}\right)\right)\nu \left(G_{z}\right)\right\}. \end{array}$$

For the developments presented in Section 3, we focus on two-dimensional (2D) *G*-space (latitude and longitude), but the concepts presented here could naturally extend to other dimensions like time (1D) or 3D, when altitude is included. The *D*-space dimension is set as 2, as in Section 3.1 and Section 3.2, balancing deformation and computational complexity.

The interpolation of the process *d* at coordinates $s_{W}\;:=\left[\begin{array}{ccc} {\tilde{s}}_{1} & \ldots & {\tilde{s}}_{W} \end{array}\right]$, ${\tilde{s}}_{w}\in G$, ∀w=1,…,W may be required. This is carried out by applying the standard conditional distribution properties of multivariate normal combined with matrix-variate and multivariate normal relationships (e.g., Gupta and Nagar [28]). Suppose

$$\begin{array}{c} \left[\begin{array}{c} vec(d_{W})\\ vec(d_{Z}) \end{array}\right]∼\mathrm{MN}\left(\left[\begin{array}{c} vec(s_{W})\\ vec(s_{Z}) \end{array}\right],\left[\begin{array}{cc} R_{11} & R_{12}\\ R_{21} & R_{22} \end{array}\right]⊗\sigma_{d}^{2}\right) \end{array}$$

where $d_{W}\;:=\left[\begin{array}{ccc} d({\tilde{s}}_{1}) & \ldots & d({\tilde{s}}_{W}) \end{array}\right]$; ${\left[R_{11}\right]}_{ij}=\rho_{\phi }(∥{\left[vec(s_{W})\right]}_{i}-{\left[vec(s_{W})\right]}_{j}∥)$ for i=1,…,W and j=1,…,W where ${\left[vec(s_{W})\right]}_{i}\;:={\tilde{s}}_{i}$ denotes each coordinate to be interpolated; ${\left[R_{22}\right]}_{ij}=\rho_{\phi }(∥{\left[vec(s_{Z})\right]}_{i}-{\left[vec(s_{Z})\right]}_{j}∥)$ for i=1,…,Z and j=1,…,Z, with ${\left[vec(s_{Z})\right]}_{i}\;:=s_{i}$ denoting each centroid coordinate from the partition; and ${\left[R_{12}\right]}_{ij}=\rho_{\phi }(∥{\left[vec(s_{W})\right]}_{i}-{\left[vec(s_{Z})\right]}_{j}∥)$ for i=1,…,W and j=1,…,Z; and finally $R_{21}={R_{12}}^{T}$. Therefore, the distribution of $vec\left(d_{W}\right)|vec\left(d_{Z}\right)$ is given by

$$\begin{array}{c} vec\left(d_{W}\right)|vec\left(d_{Z}\right)∼\mathrm{MN}\left(\mu_{W|Z},\Sigma_{W|Z}\right) \end{array}$$

where

$$\mu_{W|Z}=vec\left(s_{W}\right)+\left(R_{12}R_{22}^{-1}⊗I_{2}\right)\left(vec\left(d_{Z}\right)-vec\left(s_{Z}\right)\right)$$

and $\Sigma_{W|Z}=\left(R_{11}-R_{12}R_{22}^{-1}R_{12}^{T}\right)⊗\sigma_{d}^{2}$. The codes provided in Appendix A can be used to obtain a sample of size *L* from the posterior distribution of $vec(d_{W})$. Specifically, for each *l*-th sample of $vec{\left(d_{Z}\right)}^{\left(l\right)}$, a sample from the posterior distribution of $vec(d_{W})$ can be generated from:

$$\begin{array}{c} vec\left(d_{W}\right)|vec\left(d_{Z}\right)∼\mathrm{MN}\left(\mu_{W|Z},\Sigma_{W|Z}\right) \end{array}$$

where $\mu_{W|Z}^{\left(l\right)}=vec\left(s_{W}\right)+\left(R_{12}R_{22}^{-1}⊗I_{2}\right)\left(vec{\left(d_{Z}\right)}^{\left(l\right)}-vec\left(s_{Z}\right)\right)$ and the interpolation is based on the mean distribution.

Function *d* is unidentifiable, since all the transformations of the coordinates in *D*-space which keep the same distances between locations are observationally equivalent. Scaling all distances by a constant is also equivalent. For the practical application of the deformation model in the Bayesian paradigm, fixing the locations of two points in *D*-space is recommended (Damian et al. [29], Morales et al. [26], Sampson and Meiring [30]). We anchor $d\left(s_{1}\right)\;:=s_{1}$ and $d\left(s_{Z}\right)\;:=s_{Z}$ to prevent translation or rotation.

Bayesian inference is implemented via Markov Chain Monte Carlo (MCMC). Sampling of the posterior samples was handled by applying Hamiltonian Monte Carlo (HMC). Non-centered parameterizations (see, for example, Papaspiliopoulos et al. [31]) were employed for both the Gaussian process β and deformation process *d*. This choice also enhances favorable a posteriori geometry and computational efficiency. The analyses were accomplished on R version 4.0.3 (R Core Team (2020)) and some MCMC parts were developed on C++ and Rcpp library to make an interface between R and C++. The Rstan package (Carpenter et al. [32]) provided comparable results and was used to make an interface. The Stan codes used in all subsequent analyses are available in Appendix A.

## 3. Results

In this section, comparisons between our anisotropic proposal and isotropic formulations are presented, based on simulated and real data. The analyses were performed on a notebook with a Core i5 processor and 8 GB RAM. When the DP was considered, convergence times ranged from 1.5 to 4 hours, with a total number of iterations ranging from 1500 to 3000, and with burn-in ranging from 500 to 2000 and no thinning. When considering the isotropic covariance structure, convergence times ranged from 30 minutes to 1 hour. The convergence of the chains was evaluated by observing the trace plot and the statistics: effective sample size (ESS) and $\hat{R}$, available on Rstan library.

Anisotropic and isotropic formulations were fitted to both synthetic and real data. Their ability to recover patterns associated with the intensity function of the underlying point process was also evaluated. The adopted model comparison criteria consider different aspects such as predictive ability, the trade-off between fit quality and complexity, and estimation accuracy. Such criteria include the sum of the Conditional Predictive Ordinate (CPO) on the logarithmic scale (slCPO) (Pettit [33]), Deviance Information Criterion (DIC) (Spiegelhalter et al. [34]), averaged Interval Score (AIS) (Winkler [35]), and Mean Absolute Error (MAE).

### 3.1. Simulating the Process with a Deterministic Intensity

Point processes with a substantially varying intensity function favor anisotropic models. This assumption is tested with a PP with intensity function given by

(12) $$f\left(x,y\right)=\left\{\begin{array}{cc} 191\sin\left(22.8x^{2}{\left(1-y\right)}^{2}\right)e^{x^{2}+{\left(1-y\right)}^{2}}+1150 & \;y<x<1\;\;\mathrm{and}\;\;0<y<1\\ 191\sin\left(22.8x^{2}{\left(1-y\right)}^{2}\right)e^{x^{2}+{\left(1-y\right)}^{2}}+1150+450\cos\left(10y^{1.2}{\left(1-x\right)}^{1.2}\right) & \;0<x<y\;\;\mathrm{and}\;\;0<y<1 \end{array}\right.$$

**Figure 1:**
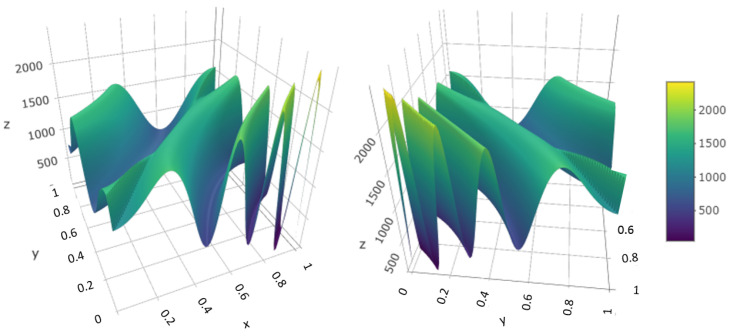
Intensity function given by expression (12) exhibited in different angles.

Figure 1 exhibits the intensity function generated by the deterministic expression (12). The anisotropy assumption is likely to be more adequate due to the varying level of oscillation of the intensity function as it gets close to the corners. The deformation should be significant and stretch the original space in the direction of the highest oscillation.

**Figure 2:**
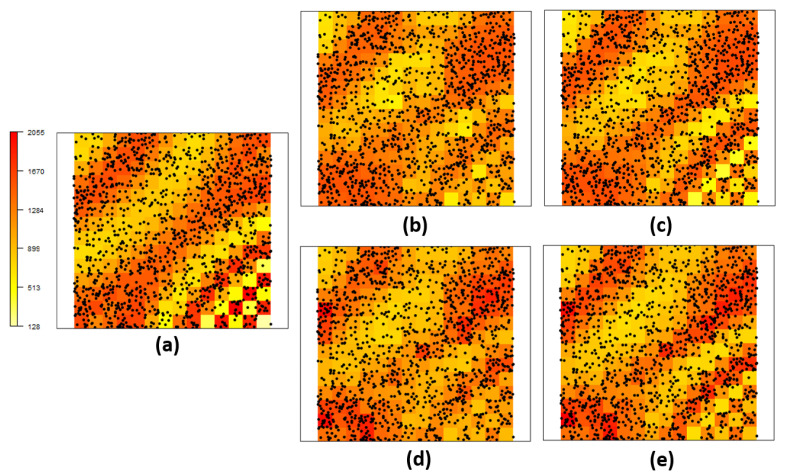
Estimation of the intensity function of expression (12): (**a**) True intensity function on the defined grid along with generated point process (dots), (**b**) estimated intensity function not considering and (**c**) considering the deformation for *probit* link function, (**d**) estimated intensity function not considering and (**e**) considering the deformation for *log* link function.

**Figure 3:**
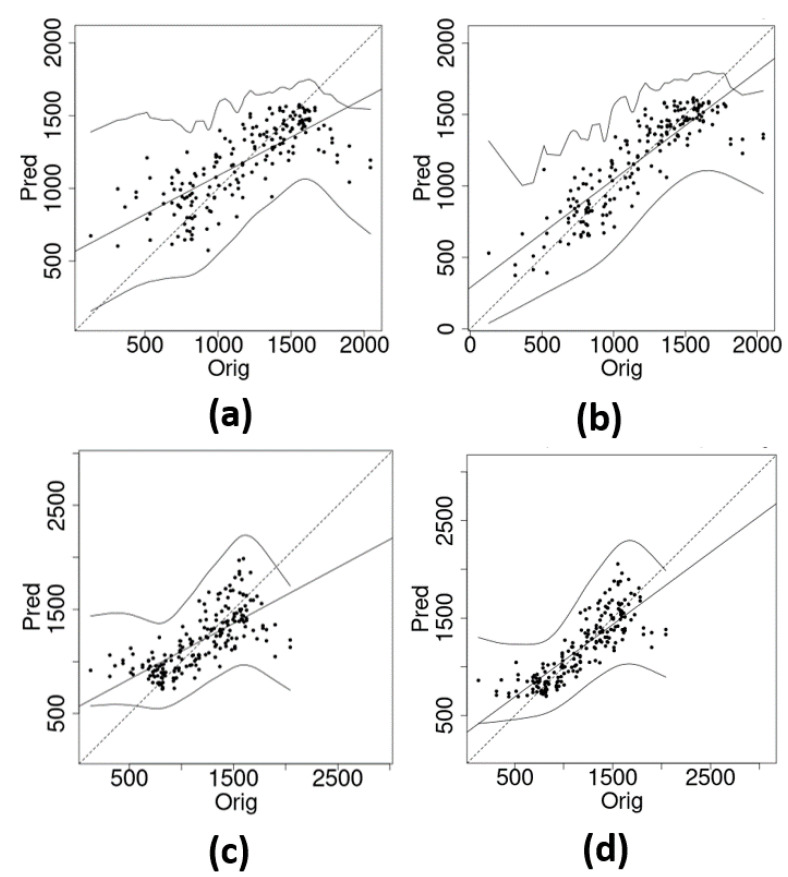
Further details of estimation of the intensity function (12). Scatter plot of true and estimated intensity function for different estimating scenarios: (**a**) intensity function not considering and (**b**) considering the deformation for *probit* link function, (**c**) intensity function not considering and (**d**) considering the deformation for *log* link function. The dots represent the pair of true and estimated intensity function values for all pixels. Full and dashed lines represent the identity and the exploratory regression lines, respectively. The latter is obtained by performing the fit of the estimated values based on the assumed true values as a covariate.

Figure 2 shows the discretized version of the original intensity function (12) and its resulting estimation with different link functions and deformation options (with/without). Panel (a) displays the true intensity function. It is clear that the estimation process considering anisotropy via DP, displayed in panels (c) and (e) (for the probit and log link functions, respectively) produced a better recovery of the intensity function patterns than the respective isotropic processes, displayed in panels (b) and (d), for the same link functions. The DP formulation performs better, especially for the region of higher variation, regardless of the choice of the link function. Figure 3 reports an exploratory analysis with a comparison between posterior intensity function point estimates along with their true values. Again, it confirms that better estimates were obtained also for the lowest and highest values for the models considering nonstationarity. The model selection criteria presented in Table 1 show that the scenarios where both the DP and the probit link function showed superior performance.

**Figure 4:**
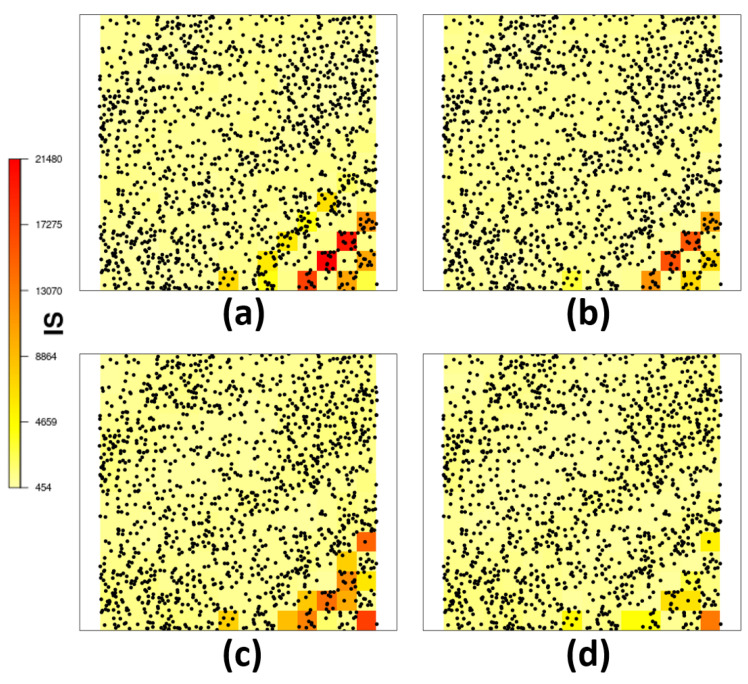
For the scenario of expression (12): (**a**) IS for the model not considering and (**b**) considering the deformation for *probit* link function, (**c**) IS for the model not considering and (**d**) considering the deformation for *log* link function.

Figure 4 shows the interval score (IS) for the estimated intensity function from scenario (12) with and without the DP for the *probit* and *log* link function. It can be noted that both models where DP was estimated seem to have smaller IS, especially for the region with higher variation for both link functions.

**Figure 5:**
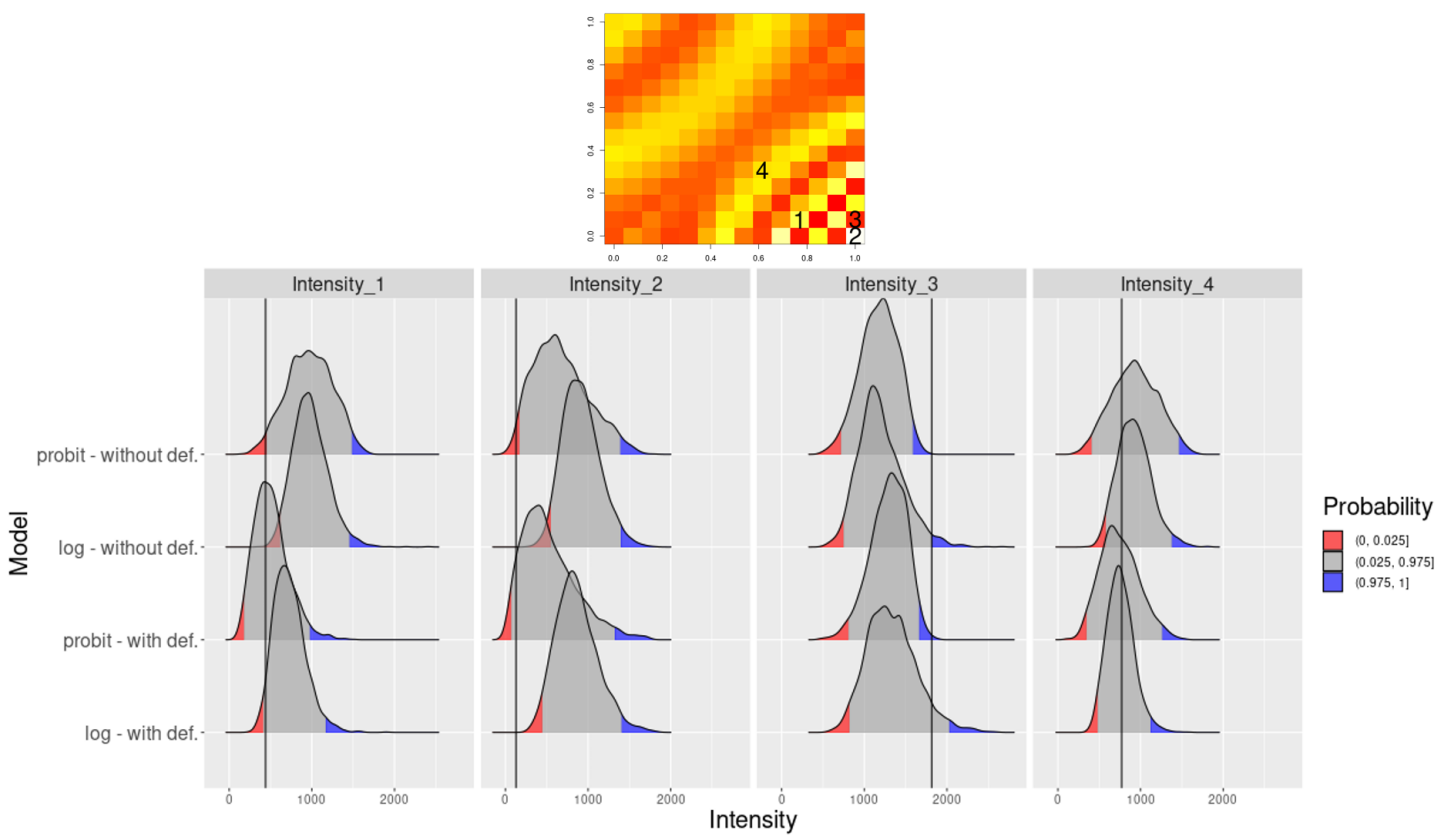
Intensity function estimation for the four selected locations: true intensity function (**top**) and density plot for the selected locations with their respective credibility interval of 95% (**bottom**) for the four models considered.

Figure 5 compares intensity function posterior density for specific locations, revealing different intensity function prediction behaviors based on intensity function regimes. Locations 1 and 2 (in the low intensity function region) show an estimated intensity function closer to true values and CI, enclosing true value only when DP is assumed. Location 3 (in high-intensity region) exhibits CI, including true intensity with *log* link and DP assumption. Location 4 (in the smooth intensity function region) shows similar results for all models.

**Figure 6:**
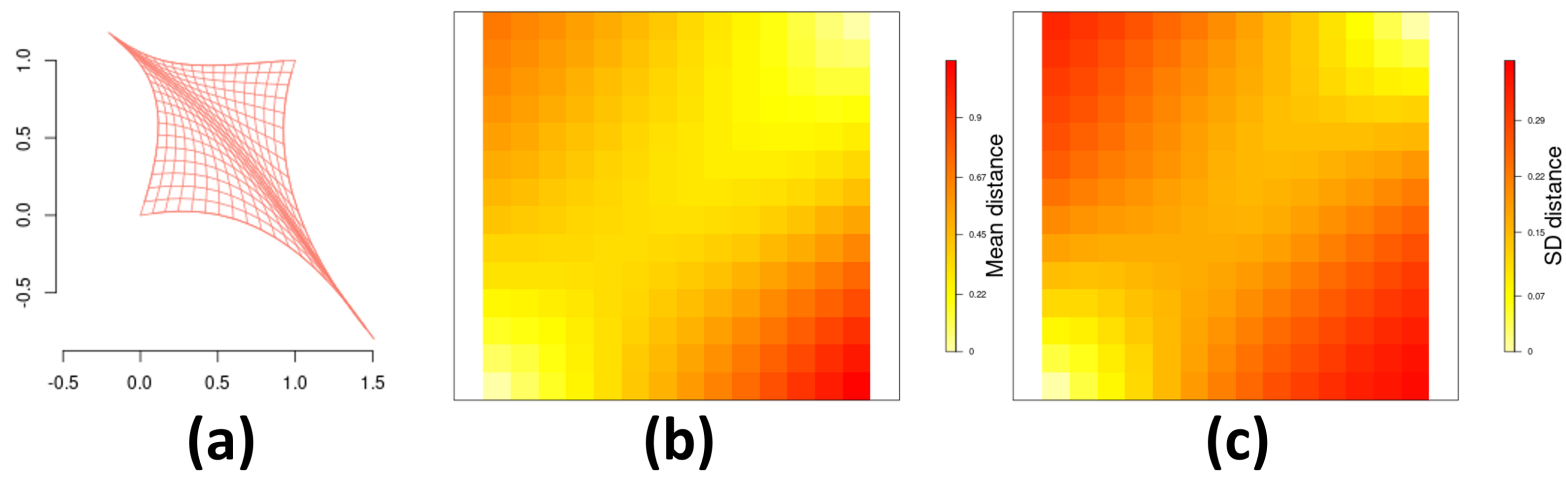
Estimation of the deformation *d*: (**a**) estimated mean deformation for the *probit* link function; (**b**) mean and (**c**) standard deviation for the posterior distribution of the distance between each centroid $s_{z}$ and $d(s_{z})$ for z∈{1,…,Z} for the *probit* link function.

In order to avoid confusion due to the differences between hyperparameters associated with different link functions, and since results show an overall superior performance of the model with probit link, further results from Figure 6 present more details for the *probit* link only. Greater deformation from the original grid (near (0,1) and (1,0)) resulted in higher SD variability. Our approach is fully Bayesian and Table 2 displays mean values and credibility intervals for hyperparameters that are estimated through the Hamiltonian Monte Carlo algorithm described in Appendix A.

A sensitivity analysis was carried out to examine the impact on estimated intensity function from fixed and estimated parameters. The analysis showed that intensity function estimates were robust to the proposed values for range ϕ, covariance $\sigma_{d}^{2}$, precision δ, and intercept μ, within plausible parameter ranges. The sensitivity to the prior distribution for range parameter θ was low, while different priors for maximum intensity $\lambda^{*}$ had some impact, warranting careful consideration. Details about sensitivity studies are available at Quintana (2022) ([36], Sections 3.5 and 4.2).

This section presented the results of inference based on data generated from an intensity function with substantial variation. The overall message from the exercise of this section is that the use of deformation improved upon inference with isotropic models. A number of different aspects were considered in this comparison, and most of them showed substantial improvement, stemming from the incorporation of anisotropy. Another result that was somewhat unexpected is that the probit link compared favourably against the frequently used logarithmic link. This effect could be associated with the versatility of the probit transformation that enables it to mimic even the logarithmic function by adapting its seemingly unbounded first half to cover the entire logarithmic trajectory.

### 3.2. Real Data Application—Spodoptera frugiperda Pest in a Corn-Producing Agricultural Area

**Figure 7:**
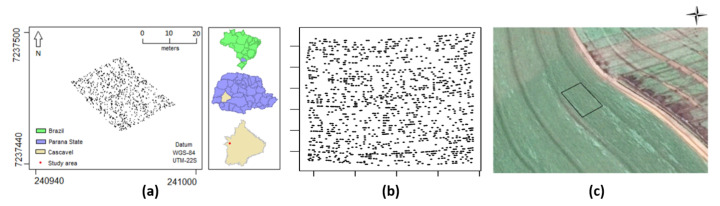
Point pattern for the corn plants infected by the *S. frugiperda* with (**a**) original and (**b**) rotated latitude and longitude and (**c**) a satellite image of the experimental area with the polygon of the study area. Adapted from Nava et al. [37].

The analyses presented in this section concern a real data experiment on the effects of a pest over a corn plantation. The data collection was carried out in an agricultural commercial area of 27 ha located in the city of Cascavel, Paraná state, Brazil (approx. 24.95° S, 53.57° W and 650 m asl). An occurrence is defined by the location of a plant of corn attacked with the fall armyworm in the experimental area during the crop year 2015/2016. The location is defined by the geographical coordinates of the infected plant, which were obtained using a GEOEXPLORE 3 GPS positioning system receiver in a Universal Transverse Mercator (UTM) coordinate system. The study area included 9360 corn plants, of which 1303 were infected by the *S. frugiperda* (Figure 7a). The dataset was provided by the Spatial Statistics Laboratory of Unioeste.

Nava et al. [37] investigated the dominant infestation behavior of fall armyworm in that agricultural area. They assessed whether the point pattern showed aggregation or regularity using tests proposed by Guan et al. [38], Mateu and Nicolis [39] and Rajala et al. [7] to detect anisotropic spatial behavior. They found significant anisotropy in the original dataset, detected mainly along the 135- and 45-degree directions due to planter machine design and area shape. In a central subset after rotation, no dominant directions were detected, suggesting potential localized anisotropy near experimental area borders.

Our methodology differs from Nava’s approach in several ways. Firstly, their tests are limited to predefined angles, while our method adapts to structures at any angle or even different angles in various geographic regions. Secondly, their method evaluates geometric anisotropy, while ours applies to a broader class of anisotropy. Most importantly, while their approach tests indicative angles of anisotropy, our method estimates an intensity function that fully accounts for any kind of data-driven anisotropy.

**Figure 8:**
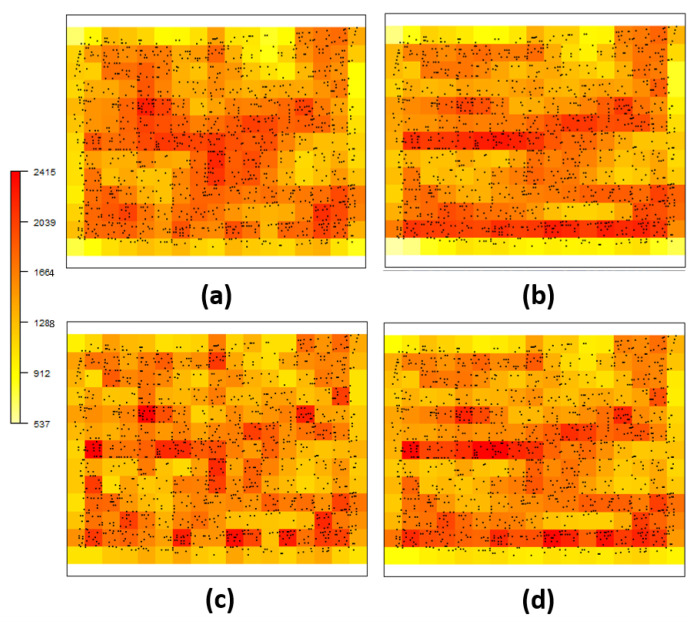
Estimation of the intensity function for *Spodoptera frugiperda* pest data: (**a**) not considering and (**b**) considering the deformation for probit link function, (**c**) not considering and (**d**) considering the deformation for log link function.

Figure 8 displays infected plant locations and the estimated intensity function on a 17×13 grid for different link functions and DP considerations, while Table 3 shows the results of model comparison criteria. Our anisotropic formulation via DP shows better performance over the corresponding model without deformation. Additionally, the *probit* link function seems to provide better results over the corresponding model with logarithmic link.

Intensity function estimation with DP showed similar behavior for both link functions, as shown in Figure 8 (items (b) and (d)). Bands appeared near the horizontal direction, showing similarity in higher or lower intensity values. When stationarity/isotropy was assumed and DP was not used (items (a) and (c) in Figure 8), the intensity function appeared less smooth, lacking clear intensity bands horizontally.

**Figure 9:**
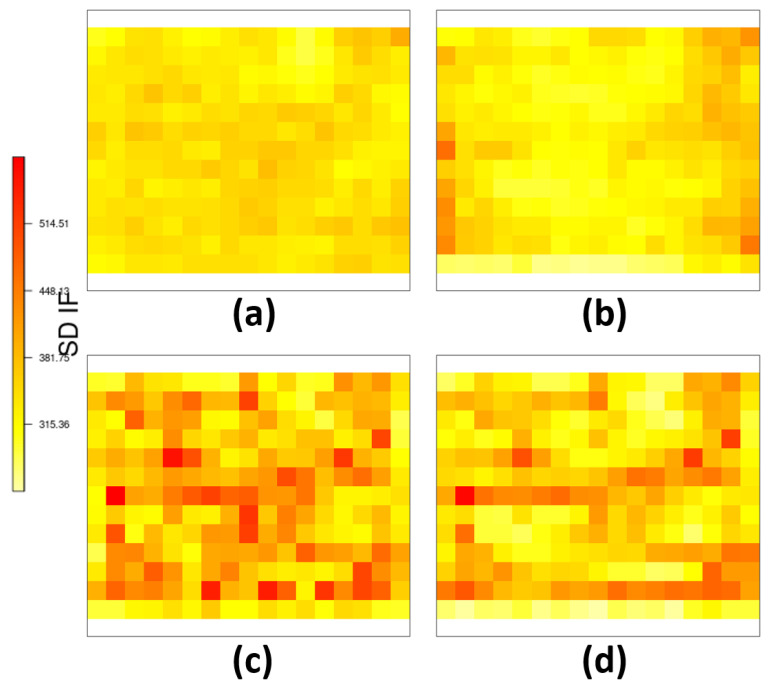
Standard deviation for the intensity function for the model for the *Spodoptera frugiperda* pest data: (**a**) not considering and (**b**) considering the deformation for *probit* link function, (**c**) not considering and (**d**) considering the deformation for *log* link function.

Figure 9 displays the posterior standard deviation of intensity function for *Spodoptera frugiperda* pest data for all considered models. The *probit* link showed almost uniformly lower SD in intensity function estimates compared to the *log* link. Higher posterior SD for the intensity function along with higher SD variability were observed with deformation when the *log* link was considered.

**Figure 10:**
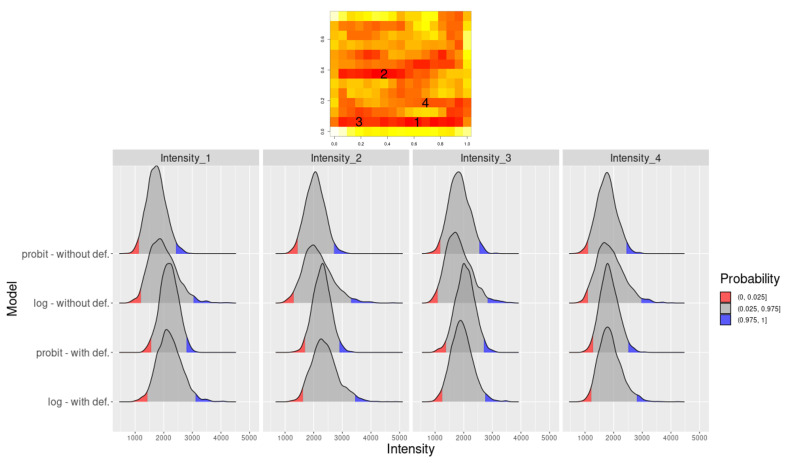
Estimated intensity function map for the *Spodoptera frugiperda* pest data showing the positions of intensity function selected for posterior density comparison across models (**top**) and density plot for the selected positions with their respective credibility interval of 95% (**bottom**).

Figure 10 compares intensity function posterior density for specific locations by evaluating different intensity function predictions in areas with varying intensity. Locations #1, #2, #3 and #4 showed more concentrated posterior distributions for models considering DP when compared to the corresponding isotropic models. Table 4 displays *log* CPO values, indicating better predictive capacity in these regions for DP models.

**Figure 11:**
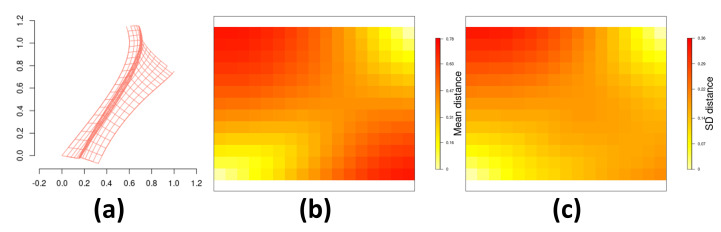
Estimation of the deformation *d* for the *Spodoptera frugiperda* pest data: (**a**) estimated mean deformation for the *probit* link function; (**b**) mean and (**c**) standard deviation for the posterior distribution of the distance between each centroid $s_{z}$ and $d(s_{z})$ for z∈{1,…,Z} for the *probit* link function.

Figure 11 depicts the DP-estimated intensity function surface and the posterior mean and standard deviation for the distance between centroids $s_{z}$ and $d(s_{z})$ for the *probit* link function. These statistics help assess uncertainty related to the estimated DP. We can see a compressing space near the horizontal direction, suggesting higher spatial correlation in that orientation. The bands detected in the intensity function also align with this correlation. Variability in estimates increased in regions farther from the original grid, observed in the SD plot.

Table 5 displays the mean values and credibility intervals for parameters ($\lambda^{*}$, θ) when *probit* link function is considered. Although there are differences in posterior point estimates, the associated posterior uncertainty in both cases prevents any distinction between the resulting inference for these hyperparameters.

**Figure 12:**
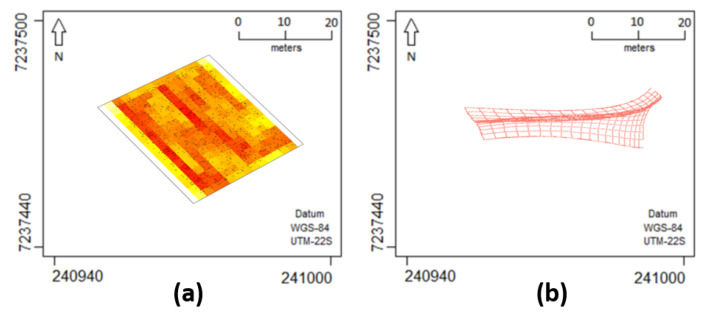
Estimated (**a**) intensity function and (**b**) DP for the model considering the *probit* link function for unrotated and rescaled *Spodoptera frugiperda* pest data.

Finally, Figure 12 illustrates the estimated intensity function and DP using the best fit scenario (*probit* link function with DP estimation) on the original, unrotated, and rescaled geographic region. Overall, this real data analysis showed results that coincide qualitatively with those from the analysis of synthetic data. Namely, in the presence of intensity functions with high variability, models incorporating deformation outperformed the corresponding isotropic versions across a variety of comparison criteria.

### 3.3. Comparative Analysis of Spatial Deformation and Geometric Anisotropy

As mentioned in Section 1, alternative approaches exist to address the anisotropy of spatial processes, with geometric anisotropy being a commonly used methodology (see, for example, ref. [40]). This approach is relatively straightforward to implement, involving a linear transformation corresponding to the rotation and stretching of the geographic coordinate axes, and relies solely on the estimation of two parameters: the rotation angle and the anisotropic ratio between the axes. However, it restricts anisotropy to a predefined structure. This section compares several model selection criteria for the model considering geometric anisotropy with the proposed model that incorporates spatial deformation to address the inherent anisotropy in the data for both *probit* and *log* link functions.

For the simulated process shown in Section 3.1, the estimated rotation angle was $-43.23^{\circ }$ and the anisotropic ratio was 1.41 when geometric anisotropy was considered. The model selection criteria presented in Table 6, comparing the results for the model considering geometric anisotropy and the model considering spatial deformation for both *probit* and *log* link functions, indicate that the scenarios where both spatial deformation and the *probit* link function were used showed superior performance.

For the real data analysis presented in Section 3.2, the estimated rotation angle was $3.27^{\circ }$, and the anisotropic ratio was 1.21 when geometric anisotropy was considered. The model selection criteria shown in Table 7, which compare the results of the model considering geometric anisotropy with the model considering the DP for both *probit* and *log* link functions, again demonstrate that the scenarios where both the DP and the *probit* link function were employed exhibited superior performance.

## 4. Discussion

The proposed point process analysis model, incorporating anisotropy and nonstationarity via DP, outperformed models lacking these considerations in both simulated and real data analyses. Criteria assessing fit quality showed marked improvements. Specifically, in a scenario observing varying oscillation frequencies, models accommodating deformation showed better discrimination between higher and lower frequency zones than those without. Models omitting deformation failed to distinguish the intensity regions accurately. Incorporating deformation notably enhanced all model selection criteria, favoring the model that accounted for it. This approach, addressing anisotropy and nonstationarity via spatial deformation (rather than other specific pattern across the entire space), proved beneficial because the change in the intensity function regime occurred mainly in the relevant subregions, by deforming the geographic space differently across regions. Moreover, specifying a parametrized anisotropy and/or nonstationarity formulation for these intensity function behavior would be insufficient to capture these types of changes in the intensity function. Comparisons between the proposed anisotropic model via deformation and the geometric anisotropic model indicated favorable results for the proposed formulation. This was observed in both artificially generated data and real data concerning the *Spodoptera frugiperda* pest.

Deformation behavior in *Spodoptera frugiperda* pest control data shows increased spatial correlation along the horizontal direction (in rotated coordinates), aligning with findings from Nava et al. [37], highlighting higher anisotropy at a $135^{\circ }$ angle in non-rotated data. This is consistent with agricultural practices, mirroring the direction of planting machines. Estimated infestation levels enable targeted pesticide application, aiding environmental and consumer health by prioritizing doses in specific regions and prevailing directions.

Different link functions may suit each case, especially when assessing the behavior of the intensity function. In scenarios with low overall intensity but sudden spikes, the logarithm link function can better capture peaks. Alternatively, the *probit* link function might perform better for frequent sudden intensity function decays. When the intensity function behaves erratically, fitting both and selecting the best performance based on model criteria proves relevant in practical terms.

An extended model includes covariates influencing the point pattern by substituting {β(s):s∈G} with {W(s)β(s):s∈G} in expression (3), where W(s) represents some form of square root precision matrices. Similarly, for some applications offsets {r(s):s∈G} might be needed for a comparison of varying population sizes across space. For instance, defining Λ(s) :=λ(s)r(s) in expression (3) allows the data *Y* to be rewritten as Y|Λ∼PP(Λ).

The isotropic model can be obtained as the special case of our DP when the main diagonal of $\sigma_{d}^{2}$ tends to zero. Model comparison may then be also obtained by a mixture model with prior weight α for isotropy and complementary prior weight 1−α for the DP component. Posterior inference on α might prove useful for assessing the relevance of the anisotropic component.

The work of this paper considered only discretized space. Recent sampling methods using Poisson thinning offer crucial extensions towards inference in continuous space. Gonçalves and Gamerman [25] utilized Poisson thinning for non-homogeneous Poisson processes, employing a spatial Cox model driven by a Gaussian process. Their MCMC algorithm allowed for direct simulation, resembling our model with a *probit* link function. Extending the methods in this direction merges the benefits of continuous surfaces, improves the intensity estimation for nonstationary/anisotropic processes, and are the subject of our interest for future work.

## Figures and Tables

**Table 1:** Some model selection criteria comparing the estimation of the intensity function for the simulated data (12) considering or not considering the deformation using the *probit* or the *log* link function.

Link	Deformation	MAE	AIS	slCPO	DIC
***probit***	without	180.76	1463.83	−507.68	988.25
	with	**133.5**	**1163.06**	**−479.31**	**947.21**
*log*	without	199.43	1588.27	−508.09	1001.09
	with	154.67	1198.28	−484.63	966.1

**Table 2:** The estimated mean and 95% credibility interval (CI) for the maximum intensity $\lambda^{*}$ and range parameter θ for the simulated data (12) considering or not considering the deformation using the *probit* link function.

Deformation	$\hat{\lambda^{*}}$ (CI$95\%(\lambda^{*})$)	$\hat{\theta }$ (CI95%(θ))
without	1646.7 (1464; 1889.6)	0.51 (0.21; 1.11)
with	1697.4 (1518.5; 1933.2)	0.65 (0.28; 1.36)

**Table 3:** Model selection criteria comparing the estimation of the intensity function for the *Spodoptera frugiperda* pest data for all models considered.

Link	Deformation	slCPO	DIC
***probit***	without	−550.584	1075.75
	with	**−537.145**	**1055.69**
*log*	without	−553.976	1087.62
	with	−543.871	1069.05

**Table 4:** Estimated intensity function and CPO (in log scale) for locations of Figure 10.

Deformation	Link	Mean Intensity Function(loc[1])	lCPO[loc[1]]	Mean Intensity Function(loc[2])	lCPO[loc[2]]
without	probit	1743.21	− 4.03	2048.23	−3.54
	log	1973.68	−3.72	2144.00	−3.93
with	probit	2187.37	−2.68	2285.29	−2.90
	log	2205.25	−2.94	2392.56	−2.99
Deformation	Link	mean intensity function(loc[3])	lCPO[loc[3]]	mean intensity function(loc[4])	lCPO[loc[4]]
without	probit	1840.86	−2.56	1754.79	−3.35
	log	1821.05	−2.75	1882.98	−3.27
with	probit	2048.01	−2.28	1846.86	−2.91
	log	1939.08	−2.43	1876.19	−3.00

**Table 5:** The estimated mean and credibility interval (CI) of 95% for the maximum intensity $\lambda^{*}$ and range parameter θ for the *Spodoptera frugiperda* pest data considering or not considering the deformation using the *probit* link function.

Link	Deformation	$\hat{\lambda^{*}}$ (CI$95\%(\lambda^{*})$)	$\hat{\theta }$ (CI95%(θ))
*probit*	without	3648.1 (2895.8; 4526.0)	1.88 (0.77; 4.01)
	with	3698.7 (2993.7; 4506.2)	1.71 (0.68; 3.59)

**Table 6:** Some model selection criteria comparing the estimation of the intensity function for the simulated data (12) considering the geometric anisotropy and the DP using the *probit* or the *log* link function.

Link	Anisotropy	MAE	AIS	slCPO	DIC
**probit**	Geometric	198.82	1529.01	− 505.94	985.27
	Deformation	**133.5**	**1163.06**	**−479.31**	**947.21**
*log*	Geometric	205.71	1639.04	−507.24	997.5
	Deformation	154.67	1198.28	−484.63	966.1

**Table 7:** Model selection criteria comparing the estimation of the intensity function for the *Spodoptera frugiperda* pest data considering the geometric anisotropy and the DP using the *probit* or the *log* link function.

Link	Anisotropy	slCPO	DIC
***probit***	Geometric	−548.351	1072.98
	**Deformation**	**−537.145**	**1055.69**
*log*	Geometric	−554.508	1087.49
	Deformation	−543.871	1069.05

## Data Availability

Simulated data is freely available upon request from author MSBQ and real data is freely available from Unioeste’s Spatial Statistics Laboratory—Campus Cascavel upon request.

## Abbreviations

    The following abbreviations are used in this manuscript:

IS	Interval score
AIS	Averaged interval score
CI	Credibility interval
CPO	Conditional predictive ordinate
DIC	Deviance information criterion
DP	Deformation process
GP	Gaussian process
IS	Interval score
HMC	Hamiltonian Monte Carlo
LGCP	Log–Gaussian Cox process
MAE	Mean absolute error
MCMC	Markov Chain Monte Carlo
PP	Poisson process
slCPO	Sum of the *log* of the conditional predictive ordinate
SD	Standard deviation
